# Dosimetric Comparison between the HyperArc and Conventional VMAT in Cervical Spine Stereotactic Radiosurgery

**DOI:** 10.3390/jcm13185497

**Published:** 2024-09-17

**Authors:** Jeehoon Park, Byungdo Park, Jeongho Kim

**Affiliations:** Department of Radiation Oncology, Samsung Changwon Hospital, Sungkyunkwan University School of Medicine, Changwon 630-522, Republic of Korea

**Keywords:** HyperArc, cervical spine, stereotactic radiosurgery, stereotactic radiosurgery normal tissue objective

## Abstract

**Background:** This research aims to evaluate the usability of the HyperArc (HA) technique in stereotactic radiosurgery for cervical spine metastasis by comparing the dosimetry of the target and organs at risk, specifically the spinal cord, between HA and VMAT and conventional volumetric modulated arc therapy (VMAT). **Methods:** A RANDO^®^ phantom and QFix Encompass^TM^ and support system were used to simulate three target types (A, B, and C) based on RTOG0631 guidelines. Treatment plans included one VMAT and two HyperArc techniques with different SRS NTO values (100 and 250). Dosimetric parameters such as conformity index (CI), homogeneity index (HI), R_50_, and spinal cord sparing were analyzed. Gamma analysis was performed using portal dosimetry to validate the dose delivery accuracy. **Results:** HyperArc plans demonstrated higher conformity, sharper dose fall-off, and improved quality assurance (QA) results compared to VMAT plans. HA with SRS NTO 250 showed even better results in terms of conformity, dose fall-off, and spinal cord dose reduction (V_10_ and D_max_) compared to HA with SRS NTO 100. Although the mean gamma passing rates were slightly lower, all plans achieved rates above 95%. **Conclusion:** The findings suggest that HA provides superior dosimetric benefits over VMAT and could be effectively utilized for cervical spine radiation therapy.

## 1. Introduction

The spine is one of the sites highly susceptible to metastasis from primary cancer. Such spinal metastases can induce severe pain in patients and lead to neurological symptoms due to compression of the spinal cord by tumors [1]. Therefore, not only curative radiation therapy, but also radiation therapy is crucial for pain control [1]. The treatment of metastatic spinal tumors can play a significant role in pain management and alleviate neurological symptoms caused by spinal cord compression [1]. Radiation therapy is a non-invasive treatment modality for spinal metastases, of which treatment options include three-dimensional conformal radiation therapy (3D CRT), stereotactic body radiation therapy (SBRT), and stereotactic radiosurgery (SRS) [2]. Among these, techniques that concentrate high doses of radiation on the tumor, such as SBRT or SRS, are more effective in reducing pain [3]. By delivering high doses to the tumor while minimizing the dose to normal tissues, these methods can make significant contributions to pain relief and reduce side effects in patients [4,5].

Volumetric-modulated arc therapy (VMAT) is a commonly used treatment approach for spine SRS. It can be applied to almost all areas without limitations in the treatment site. Additionally, VMAT allows for conformal dose distribution and the protection of normal tissues. VMAT is generally preferred over conventional intensity-modulated radiation therapy (IMRT) due to better homogeneity index (HI), conformity index (CI), lower monitor units (MU), and shorter treatment time [6].

Recently, Varian Medical Systems has developed a automated non-coplanar volumetric-modulated arc therapy technique called HyperArc (HA) to meet the demands of SRS dose delivery [7]. HA comes with special functions, including minimal workload, automated settings for the location of the isocenter, non-coplanar beam arrangement, and collimator angles [7]. Furthermore, with embedded stereotactic radiosurgery normal tissue objective (SRS NTO) and automatic lower dose objective (ALDO) algorithms, HA technology presents advantages in target and normal tissues. SRS NTO ensures a steep dose fall-off around the targets and prevents dose bridging between them. ALDO ensures superior planning target volume (PTV) coverage levels, with better sparing of organs at risk, compared to other technologies for radiosurgery [8].

In the early adoption of HA, it was applied to reduce the radiation dose to the normal brain in the radiosurgery of brain metastases or to decrease the dose to the hippocampus in whole-brain radiation therapy [7,8,9]. Recently, studies have delved into the application of HA not only in the context of brain tumors, but also in other anatomical sites. Pokhrel et al. reported promising dosimetric outcomes for the PTV and organs at risk (OAR) specifically in head and neck cases [10].

In the context of SRS to spine metastasis, achieving a steep dose gradient is crucial, considering the spinal cord tolerance and its spatial relationship with the target. Moreover, the presence of a concave shape in the high-dose region is essential to achieve sufficient dose coverage on the target [11]. The characteristics of the HA technique are anticipated to align well with the requirements of cervical SRS.

The aim of this research is to assess the feasibility of implementing the HA technique in single-fraction SRS for cervical spine metastasis. This will be achieved through a dosimetric comparison of the target and the spinal cord, as well as patient-specific quality assurance (QA), between conventional VMAT and HA. Furthermore, this study aims to evaluate the effectiveness of the SRS NTO by varying the numerical values.

## 2. Materials and Methods

### 2.1. Target and Spinal Cord Delineation

In this study, a RANDO^®^ phantom (The Phantom Laboratory, Salem, NY, USA) was utilized instead of patients, and a Discovery CT590 RT CT Simulator (CT, GE Healthcare, Milwaukee, WI, USA) simulation was conducted using the Encompass^TM^ and support system (QFix, Avondale, PA, USA) for the implementation of the HA technique. The slice thickness of the CT scans was 1.25 mm. For the implementation of the HA technique, we chose two specific regions: one where the isocenter could be accurately positioned at the center of the target, and another location where the isocenter could be offset but was still within the coverage of the field size. In this study, the third cervical vertebra (C3) and the seventh cervical vertebra (C7), which is the lowest cervical vertebra, were selected as the target sites. By including C7 as a target, we can provide information on the maximum treatment range of HA for cervical spine treatments.

**Figure 1:**
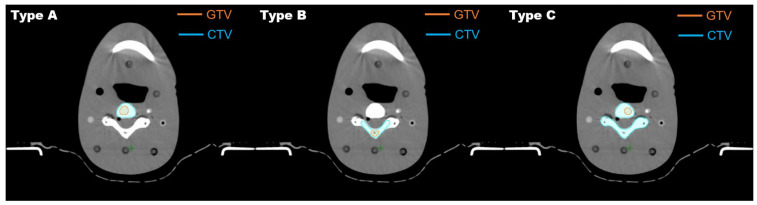
Target delineation for Types A, B, and C.

Referring to the RTOG 0631 guidelines, the gross tumor volume (GTV) and clinical target volume (CTV) were delineated into three types (Figure 1). In the first type (Type A), a segment of the spine body, which is prone to metastasis, was designated as the GTV. The CTV encompassed the entire spine body and both pedicles. In the second type (Type B), the GTV included a portion of the spine body and one pedicle. The CTV comprised the vertebral body, both pedicles, both transverse processes, spinous process, and laminae, covering the entire spine. In the third type (Type C), the GTV consisted of a portion of the spinous process and laminae. The CTV encompassed the complete spinous process and laminae. The PTV of CTV and GTV are P-CTV and P-GTV, respectively. The PTV margin was set to 2 mm for both the GTV and CTV.

The spinal cord was delineated with a 6 mm margin above and below the target volume. In the context of actual patient treatment at our institution, T1-weighted and T2-weighted images were obtained to delineate the GTV, CTV, and spinal cord. However, due to the use of a phantom in this study, the delineation of the target volume and spinal cord volume was performed without magnetic resonance imaging (MRI).

### 2.2. Treatment Planning

The Varian Eclipse treatment planning system (Version 16.0, Varian Medical Systems, Palo Alto, CA, USA) was employed in this study. The same dose constraints were applied to all plans. VMAT utilized two full arcs (coplanar) and utilized the 100 automatic normal tissue objective (NTO). HA utilized one full arc (coplanar) and three partial arcs (non-coplanar, couch angles of 45, 315, and 270). SRS NTO is an algorithm that ensures a steep dose fall-off around the targets and prevents dose bridging between them [8]. In this study, we compared the quality of plans by applying a default value of 100 and a value of 250, which is less than the priority applied to the targets. Constraints on the maximum dose of the PTV cannot be used with the ALDO algorithm. In this study, to ensure excellent PTV coverage using the ALDO algorithm, we applied constraints to the maximum dose of the body instead of the maximum dose of the target. All plans employed a 6MV-FFF beam with a dose rate of 600 MU/min. The prescription dose was delivered using the simultaneous integrated boost (SIB) technique, with the P-GTV receiving 18 Gy in a single fraction and the P-CTV receiving 10 Gy in a single fraction. The prescription dose aimed to cover 95% of the target volume. The dose constraint for the spinal cord was set to <10 Gy for 10% of its volume and <14 Gy for 0.035 cc volume (maximum dose, D_max_) [12]. The anisotropic analytical algorithm (AAA) and photon optimizer (PO) algorithm were used for all of the plans.

### 2.3. Dosimetric Comparison

The dosimetric parameters for the target include the CI, HI, and R_50_. The CI is a metric used to assess the degree of conformity between the prescription dose and the target volume. It is calculated as follows [7]:

$$CI=\frac{{TV}_{PIV}\times {TV}_{PIV}}{TV\times V_{RI}}$$

where TV_PIV_ is the volume of the target enclosed by the reference isodose volume, TV is the target volume, and V_RI_ is the reference isodose volume. The HI quantifies the uniformity of the dose distribution within the target volume. It is calculated as follows [6]:

$$HI=\frac{D_{2\%}-D_{98\%}}{D_{50\%}}$$

where D_2%_ is the near-maximal dose, D_98%_ is the near-minimal dose, and D_50%_ is the median dose. In addition, the R_50_ value was determined to evaluate the gradient index (GI) by measuring the volume of the 50% isodose region (V_50%_) [13].

$$R_{50}=\frac{V_{50\%}}{TV}$$

The gamma analysis was conducted on the TrueBeam STx using portal dosimetry to verify the dose delivery accuracy. Gamma index criteria with variations of 3%/3 mm, 2%/2 mm, and 2%/1 mm, along with threshold doses of 10%, were employed in the analysis. Tolerance of the gamma passing rate (%GPs) was 95%. Statistical significances of differences between the techniques were assessed using the Wilcoxon signed-rank test (SPSS^®^ Statistics, Version 19, Chicago, IL, USA). A two-sided *p*-value of <0.05 was considered statistically significant.

## 3. Results

**Figure 2:**
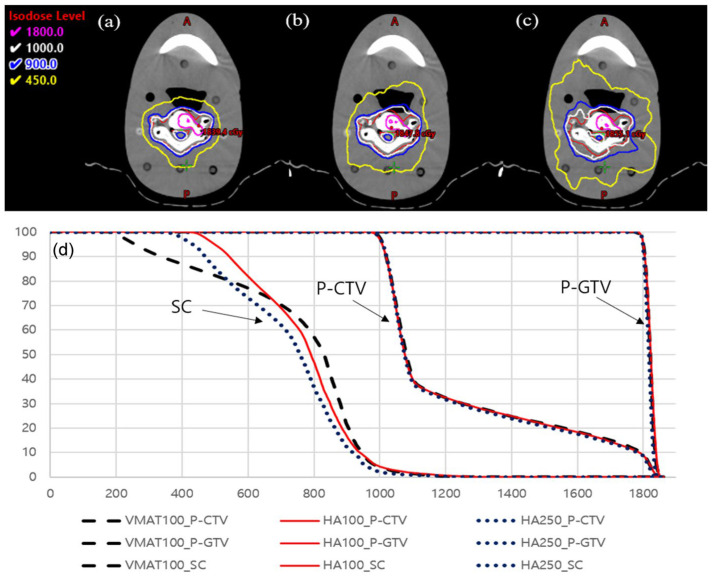
Isodose curve for (**a**) HA (SRS NTO 250), (**b**) HA (SRS NTO 100), (**c**) VMAT (NTO 100), and (**d**) DVH.

Figure 2 displays the axial view images and dose volume histogram (DVH) under different conditions. While all of the plans demonstrated similar target coverage, the HA plan showed superior spinal cord sparing when SRS NTO was set to 250. Particularly, it exhibited excellent performance in the low-dose region compared to the other two plans.

### 3.1. Dosimetric Comparision between HA (SRS NTO 100) and VMAT (NTO 100)

**Figure 3:**
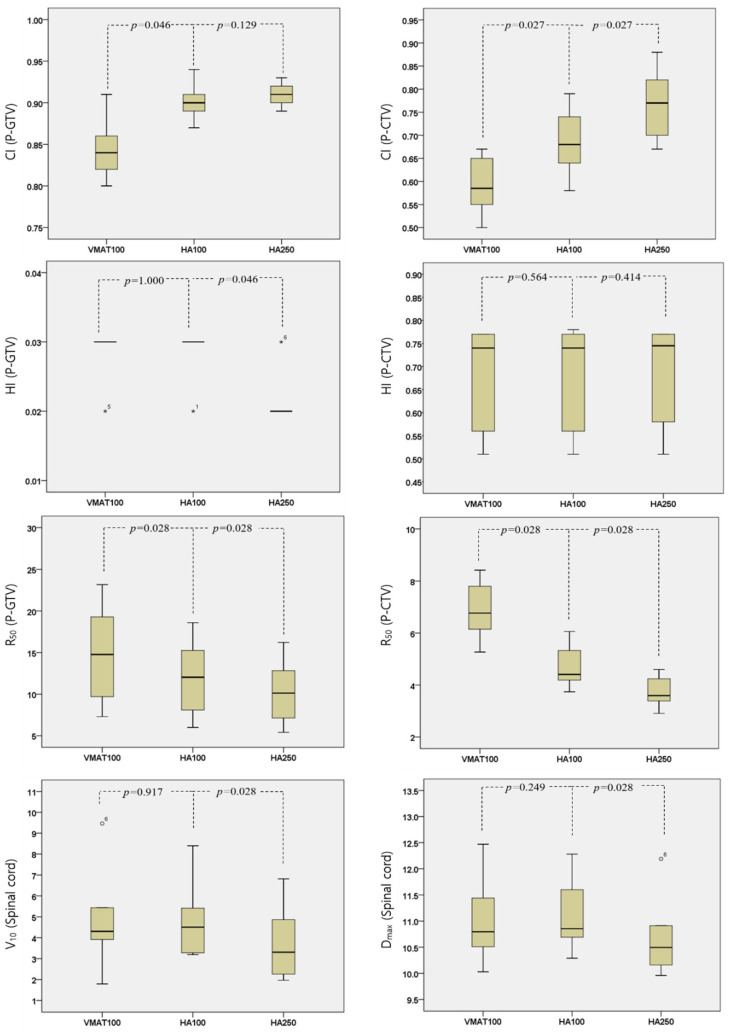
Dosimetric results for the target and spinal cord. The asterisks and circles represent outlier values, while the numbers denote the sample numbers.

Figure 3 and Table 1 present the dosimetric results of each plan. For P-GTV, the mean CI of the HA plans (SRS NTO 100) was observed to be 0.90 ± 0.03, which is 0.05 higher than the mean CI of the VMAT plan (*p* = 0.046). When comparing each plan, it was noted that all plans, except for Type B at C7, exhibited superior conformity. Both the HA and VMAT plans had a mean HI of 0.03 (*p* = 1.000), indicating a homogeneous dose distribution. Furthermore, the mean R_50_s for the HA and VMAT plans were 12 ± 4.2 and 14.83 ± 5.39, respectively, indicating that the HA plan achieved a more pronounced dose fall-off (*p* = 0.028).

For P_CTV, the mean CIs of the HA and VMAT plans were determined to be 0.69 ± 0.07 and 0.59 ± 0.06, respectively, indicating that the HA plans exhibited superior conformity (*p* = 0.027). When comparing each plan individually, it was observed that all plans demonstrated more conformity with the HA plan. The mean HI remained consistent at 0.68 for both plans (*p* = 0.564), while the mean R_50_ value for the HA plan was 4.69 ± 0.78, which was lower than the mean R_50_ value of 6.86 ± 1.04 for the VMAT plan (*p* = 0.028).

Table 2 presents the dosimetric results for the spinal cord. In the HA plan, the mean V_10_ of the spinal cord was determined to be 4.88 ± 1.76%, which was 0.01% higher than that of the VMAT plan (*p* = 0.917). Additionally, the mean D_max_ was measured as 11.10 ± 0.6 Gy, indicating a 0.1 Gy increase compared to the VMAT plan (*p* = 0.249). Although the differences were minor, it was observed that the HA plan resulted in a slightly higher spinal cord dose compared to the VMAT plan.

Table 3 presents the patient-specific QA results for each plan. In the HA plan, the mean %GPs were 97.62 ± 1.19% for the 2%/1 mm gamma index criteria, 99.72 ± 0.18% for the 2%/2 mm criteria, and 100% for the 3%/3 mm criteria. The mean %GPs were 95.78 ± 1.26% for the 2%/1 mm criteria, 99.38 ± 0.41% for the 2%/2 mm criteria, and 99.92 ± 0.07% for the 3%/3 mm criteria for the VMAT plan. It was observed that the HA plan consistently exhibited higher mean %GPs across all criteria. Furthermore, it was found that a significant number of plans in the VMAT group did not pass the gamma index criteria with a tolerance of 2%/1 mm.

The mean MU for HA plans was 4262.5 ± 10.61, which was 437.72 higher than the mean MU for VMAT plans.

### 3.2. Dosimetric Comparision between HA (SRS NTO 100) vs. HA (SRS NTO 250)

When applying SRS NTO 250, the mean CI, HI, and R50 for P-GTV were found to be 0.91 ± 0.01, 0.02, and 10.31 ± 3.56, respectively (CI *p* = 0.129; HI *p* = 0.046; R_50_ *p* = 0.028, respectively), demonstrating superior dosimetric results compared to the SRS NTO 100 HA plan. Similarly, the mean CI and R_50_ were 0.77 ± 0.07 and 3.72 ± 0.57, respectively (CI *p* = 0.027; R_50_ 0.028, respectively) for P-CTV, indicating better conformity and a steeper dose fall-off compared to the SRS NTO 100 HA plan. However, the mean HI was slightly higher at 0.69 ± 1.84 compared to the SRS NTO 100 HA plan (*p* = 0.414). Regarding spinal cord dose, the mean V_10_ was 3.75 ± 1.68%, and the mean D_max_ was 10.7 ± 0.74 Gy, both lower than the values obtained with the SRS NTO 100 HA plan (V_10_ *p* = 0.028; D_max_ *p* = 0.028) and the VMAT plan, demonstrating a lower spinal cord dose.

The mean %GPs for the different criteria were as follows: for the 2%/1 mm criteria, it was 96.37 ± 1.37%; for the 2%/2 mm criteria, it was 99.43 ± 0.25%; and for the 3%/3 mm criteria, it was 99.97 ± 0.05%. Although these values were slightly lower compared to when SRS NTO 100 was applied, all of them exceeded 95% and met the passing criteria.

When the SRS NTO was changed from 100 to 250, the mean MU increased by 340, as observed in this study.

## 4. Discussion

Bishop et al. conducted a study investigating local control, survival outcomes, and predictors of local recurrence in patients undergoing spine stereotactic body radiation therapy [14]. The study reported that poor dosimetric coverage of the GTV was associated with a higher recurrence rate [14]. Therefore, to prevent local recurrence, it is necessary to improve the coverage of the target. Our study’s results demonstrate that when applying the HA technique to cervical spine radiosurgery, it achieves superior conformity index without significantly increasing the dose to the spinal cord compared to VMAT plans. The conformity index equal to 1 corresponds to the ideal dose coverage or high conformity [15]. Thus, our study’s findings indicate that when applying HA, it results in superior dosimetric coverage compared to VMAT plans. This suggests that the application of HA is highly suitable for single-fraction spinal metastasis SRS, as demonstrated by our study results. Furthermore, the gamma passing rate of patient-specific QA for VMAT was found to be lower than that of the HA technique. In some plans, it was observed that they did not pass the criteria of 2%/1 mm criteria. This suggests that certain VMAT plans are not suitable for clinical use, as they fail to meet the required standards for treatment.

Even before the development of HA, studies employing non-coplanar VMAT were utilized in various anatomical sites, demonstrating superior target coverage and lower doses to OARs compared to coplanar VMAT [16,17,18]. Although HA and manually performed non-coplanar techniques may yield similar results, HA can utilize inherent functionalities such as protection zone and virtual dry run to prevent collisions. Additionally, it incorporates algorithms like ALDO, which enhance target coverage, and SRS NTO, which creates steep dose distributions around the target, resulting in high-quality plans that can be designed quickly and easily. Initially, these advantages were primarily utilized for brain metastasis SRS [7,8,9]. However, in recent times, they have also been applied to the treatment of head and neck or spinal metastases [10,19].

In the study by Ohira et al., it was reported that by applying HA in treatment planning for patients undergoing multifractionated spine SRS, dose coverage increased without an increase in OAR dose compared to conventional VMAT [19]. Although our study focused on single-fraction spine SRS, it exhibits remarkably similar results to Ohira’s research. However, while Ohira’s study utilized patient data, our study encompassed all forms of targets as delineated in RTOG 0631 (types A, B, C), presenting slightly more standardized outcomes. Additionally, our study conducted comparisons regarding the dosimetric results of changes in SRS NTO, a facet not explored in previous studies. SRS NTO is an algorithm that automatically generates numerous shells around the target, resulting in a steeper dose fall-off [10]. In this study, SRS NTO was set to a value of 250, which is higher than 100 and within the PTV constraint, and plans were generated and compared to plans using SRS NTO 100. The results demonstrated that using SRS NTO 250 not only provided better sparing of the spinal cord, but also resulted in significantly improved dosimetric outcomes for the target, as evidenced by measures such as CI, HI, and R_50_. However, it was observed that when using SRS NTO 250 in portal dosimetry, a lower %GPs was achieved compared to using SRS NTO 100. This can be attributed to the increased MLC modulation required to achieve a steeper dose fall-off. Nevertheless, even with the increased SRS NTO, the %GPs remained above 95% in all cases. This implies that, despite a potential decrease compared to SRS NTO 100, the treatment still satisfies all necessary criteria.

In radiation therapy, reducing intra-fractional errors and inter-fractional errors is crucial for minimizing the side effects on OARs. Since our study focused on single fractional SRS, inter-fractional errors were not considered. The HA technique offers advantages over VMAT, including steep dose fall-off and superior target coverage. However, it has the drawback of longer treatment time due to higher MU. The longer treatment times increase intra-fractional errors [20]. One approach to mitigating intra-fraction errors is to monitor patient movement during treatment. The MRIdian^®^ system (ViewRay Inc., Cleveland, OHo, USA) is a hybrid machine that consists of two main components: a 0.35 Tesla MRI scanner and a radiation delivery system, composed either of a set of three Cobalt-60 sources or a 6 MV linear accelerator [21]. These devices have the advantage of being able to monitor patient movement in real-time without delivering additional imaging doses. However, because they are designed with a bore-type configuration to acquire MR images, they are unable to perform treatment and imaging acquisition in non-coplanar fields. Another method is the use of surface-guided radiotherapy SGRT. In modern radiation therapy, the utilization of SGRT has significantly risen for enhanced patient positioning prior to online imaging and for intra-fraction motion monitoring. In numerous studies, the application of SGRT has been shown to suggest high setup accuracy and effective monitoring of intra-fractional errors, even in scenarios where open masks are employed or not [22,23,24]. The findings from these studies illustrate how SGRT can compensate for the drawback of high MU in HA plans.

This study has certain limitations. First, the contours of targets as outlined in RTOG 0631 were utilized, although there was a small number of cases. This issue will need to be addressed and refined through future clinical studies. The second limitation was the difficulty in accurately delineating the spinal cord due to the use of a RANDO^®^ phantom. However, considering that both VMAT and HA were performed under the same conditions, it is expected that comparing these two treatment plans in actual clinical data would demonstrate similar trends, as observed in this study. In order to accurately predict collisions when using the HA technique, CT scans should include the shoulders. However, in our study, we used the RANDO^®^ phantom, which limited our ability to precisely anticipate collisions. Thus, we performed protection zone verification and a dry run for each spine position. The protection zone verification results confirm that the protection zone extends up to C6, and during the dry run test, it was observed that the sixth (C6) cervical vertebrae remained collision-free. Based on these findings, the isocenter for the C3 plan was set at the center of C3, while for the C7 plan, the isocenter was positioned at the center of C6. Despite the isocenter shift for the C7 case, it was observed that better dosimetric results were achieved compared to VMAT.

## 5. Conclusions

In this study, HA and conventional VMAT plans were performed to evaluate the feasibility of applying HA in SRS treatment for the cervical spine, and dosimetric results were compared. The result was that the HA plans exhibited greater conformity to the target and a steeper dose fall-off compared to the VMAT plans. Additionally, this study compared HA plans using the default value with HA plans where the SRS NTO algorithm was increased. The results show that implementing SRS NTO 250 not only demonstrates more effective spinal cord sparing compared to applying the default value of 100, but also exhibits improved conformity to the target and a steeper dose fall-off. These results suggest that HA has utility in SRS treatment for the cervical spine. Future research should focus on applying HA to patients and evaluating its effectiveness.

## Figures and Tables

**Table 1:** Dosimetric result for the target.

Volume of Interest			Metrics	VMAT NTO 100	HA SRS NTO 100	HA SRS NTO 250
C3	Type A	P-GTV	CI	0.85	0.94	0.93
			HI	0.03	0.02	0.02
			R50	9.70	8.09	7.13
		P-CTV	CI	0.65	0.74	0.82
			HI	0.56	0.56	0.58
			R50	6.15	4.19	3.39
	Type B	P-GTV	CI	0.83	0.89	0.91
			HI	0.03	0.03	0.02
			R50	23.17	18.59	16.22
		P-CTV	CI	0.67	0.79	0.88
			HI	0.77	0.78	0.77
			R50	5.27	3.74	2.91
	Type C	P-GTV	CI	0.80	0.90	0.90
			HI	0.03	0.03	0.02
			R50	15.70	12.54	10.67
		P-CTV	CI	0.55	0.66	0.73
			HI	0.73	0.72	0.73
			R50	7.80	5.33	4.24
C7	Type A	P-GTV	CI	0.86	0.91	0.92
			HI	0.03	0.03	0.02
			R50	7.30	6.00	5.41
		P-CTV	CI	0.56	0.64	0.70
			HI	0.51	0.51	0.51
			R50	7.02	4.48	3.79
	Type B	P-GTV	CI	0.91	0.87	0.89
			HI	0.02	0.03	0.02
			R50	19.29	15.25	12.82
		P-CTV	CI	0.61	0.70	0.81
			HI	0.77	0.77	0.77
			R50	6.52	4.33	3.40
	Type C	P-GTV	CI	0.82	0.90	0.91
			HI	0.03	0.03	0.03
			R50	13.83	11.53	9.58
		P-CTV	CI	0.50	0.58	0.67
			HI	0.75	0.76	0.76
			R50	8.42	6.06	4.60

**Table 2:** Dosimetric result for the spinal cord.

Spinal Cord		Criteria	VMAT NTO 100	HA SRS NTO 100	HA SRS NTO 250
C3	Type A	V_10_ (%)	4.32	4.8	3.85
		D_max_ (Gy)	10.72	10.92	10.71
	Type B	V_10_ (%)	3.92	4.20	1.97
		D_max_ (Gy)	10.51	10.69	9.96
	Type C	V_10_ (%)	5.43	5.41	4.86
		D_max_ (Gy)	11.44	11.60	10.91
C7	Type A	V_10_ (%)	4.29	3.19	2.26
		D_max_ (Gy)	10.87	10.79	10.28
	Type B	V_10_ (%)	1.79	3.28	2.77
		D_max_ (Gy)	10.03	10.29	10.16
	Type C	V_10_ (%)	9.46	8.40	6.81
		D_max_ (Gy)	12.47	12.28	12.19

**Table 3:** Gamma pass rate result using portal dosimetry.

			VMAT NTO 100 (%)	HA SRS NTO 100 (%)	HA SRS NTO 250 (%)
C3	2%/1 mm	Type A	94.9	97.5	95.9
		Type B	94.7	98.2	96.2
		Type C	96.0	97.4	95.6
	2%/2 mm	Type A	99.4	99.7	99.2
		Type B	99.1	99.7	99.3
		Type C	99.4	99.7	99.6
	3%/3 mm	Type A	99.9	100	99.9
		Type B	99.9	100	100
		Type C	99.9	100	99.9
C7	2%/1 mm	Type A	98.2	99.5	99.3
		Type B	96.3	97.6	96.2
		Type C	94.6	95.5	95.0
	2%/2 mm	Type A	99.9	100.0	99.9
		Type B	99.8	99.8	99.2
		Type C	98.7	99.4	99.4
	3%/3 mm	Type A	100.0	100.0	100.0
		Type B	100.0	100.0	100.0
		Type C	99.8	100.0	100.0

## Data Availability

Data are contained within the article.

## Abbreviations

Anisotropic analytical algorithm (AAA); automatic lower dose objective (ALDO); automatic normal tissue objective (NTO); clinical target volume (CTV); computed tomography (CT); conformity index (CI); dose volume histograms (DVH); gamma passing rate (%GPs); gross tumor volume (GTV); head and neck (H&N); homogeneity index (HI); HyperArc (HA); intensity-modulated radiation therapy (IMRT); magnetic resonance imaging (MRI); maximum dose (Dmax); monitor units (MU); organs at risk (OAR); planning target volume (PTV); photon optimizer (PO); stereotactic body radiation therapy (SBRT); stereotactic radiosurgery (SRS); stereotactic radiosurgery normal tissue objective (SRS NTO); surface-guided radiation therapy (SGRT); three-dimensional conformal radiation therapy (3D CRT); volumetric-modulated arc therapy (VMAT); whole-brain radiation therapy (WBRT).
